# Association Between Gliflozins Use and Outcomes in Adults with Sepsis: A Multicenter Retrospective Cohort Study Among Veterans

**DOI:** 10.1016/j.aicoj.2025.100021

**Published:** 2026-01-16

**Authors:** Justine Tang, Bocheng Jing, Krystal Karunungan, Anusha Badathala, Arthur Wallace, Matthieu Legrand

**Affiliations:** a Department of Anesthesia and Perioperative Care, University of California, San Francisco, California, United States of America; b Northern California Institute for Research and Education, San Francisco, California, United States of America; c Veterans Affair Medical Center, San Francisco, California, United States of America; d Division of Geriatrics, Department of Medicine, University of California, San Francisco, California, United States of America; e Department of Epidemiology and Population Health, Stanford University, California, United States of America

**Keywords:** Sodium-glucose cotransporter-2 inhibitors, Gliflozins, Major adverse cardiovascular events, Mortality, Acute kidney injury, Critical care

## Abstract

**Background:**

Sodium-glucose-cotransporter-2 inhibitors (SGLT2i) improve cardiorenal outcomes in patients with diabetes, chronic heart failure or kidney disease, but their effects in acute settings are unknown. This study evaluated the impact of SGLT2i use on 90-day mortality, major adverse cardiovascular events (MACE), and acute kidney injury (AKI) in patients with sepsis.

**Methods:**

A propensity-matched, multicenter retrospective cohort study was conducted using the Veterans Affairs Healthcare System (VAHCS) National Registry from January 1, 2017, to December 31, 2023. Adult veterans with sepsis using SGLT2i in the year prior to admission were compared to a 1:4 matched control group, adjusting for demographics, comorbidities, medications, and sepsis characteristics. We conducted a sensitivity analysis using SuperLearner to estimate the propensity score and perform matching. Chronic SGLT2i use was defined as ≥3 outpatient fills or <180-day gap from the last fill according to the VAHCS pharmacy registries. Primary outcome was 90-day mortality; secondary outcomes included MACE within 90 days, AKI, MAKE-30, EDKA, and hospital length of stay.

**Results:**

Among 197,879 eligible patients, 5.15% were SGLT2i prior users and 94.85% were non-users. After propensity-matching, 10,200 SGLT2i users (mean [SD] age: 70.0 [9.1]; 97.5% male, and 72.4% white) were compared to 37,785 controls (mean [SD] age: 70.2 [9.4]; 97.3% male, and 72.5% white). SGLT2i prior use was associated with a significantly reduced risk of 90-day mortality (OR = 0.591, 95% CI: 0.557−0.628), AKI (OR = 0.862, 95% CI: 0.809-0.918), and MAKE-30 (OR = 0.725, 95% CI: 0.683−0.771). There was no association between SGLT2i use and MACE (OR = 0.986, 95% CI: 0.931–1.044). SGLT2i users had shorter hospital stays (13.4 vs. 18.1 days). However, SGLT2i use was associated with a significantly increased risk of EDKA (OR = 2.371, 95% CI: 2.106–2.671).

**Conclusion:**

SGLT2i users prior to hospitalization for sepsis had reduced risk of 90-day mortality and AKI, suggesting chronic organ protection decreases the risk of organ failure when sepsis develops.

## Background

Sodium-glucose cotransporter-2 inhibitors (SGLT2i), or gliflozins, have been approved by the US Food and Drug Administration (FDA) since 2013 for the management of type 2 diabetes [1]. Their indications have since expanded to include heart failure and chronic kidney disease, applicable to both diabetic and non-diabetic patients. Clinical studies have demonstrated that SGLT2i improve blood pressure regulation, glycemic control, and cardiorenal outcomes, leading to a significant reduction in mortality [[1], [2], [3]].

Patients with chronic cardiometabolic conditions are at risk of developing sepsis [4], a life-threatening host response to infection and a leading cause of acute organ failure and death in hospitalized patients. While the long-term cardiorenal benefits of SGLT2i may theoretically improve outcomes in sepsis, their efficacy and safety in the setting of acute critical illness are uncertain. SGLT2i have been associated with complications that may prove detrimental in acutely ill patients, including ketoacidosis [5] and hypovolemia due to natriuresis and osmotic diuresis [[6], [7], [8], [9], [10]]. Recent randomized trials have evaluated the use of SGLT2i in acutely ill populations. The DEFENDER trial investigated the initiation of dapagliflozin in critically ill patients with acute organ dysfunction, nearly 40% of whom had suspected infection, but found no significant improvement in the primary composite outcome [11]. In a small pilot study in cardiac surgery, SGLT2i was associated with reduced incidence of acute kidney injury [12] but the small sample size poses a high risk of error. In both trials, SGLT2i were initiated during the acute phase, and patients were exposed to treatment only for a short period of time, possibly insufficient to confer organ protection.

Whether prior exposure to SGLT2i before admission influences outcomes through chronic organ protection remains unknown. Therefore, we performed the Gliflozins and Outcomes of Adults Treated for Sepsis (GOATS) study to determine whether SGLT2i use prior to sepsis is associated with improved outcomes in adult veterans [13].

## Methods

### Study design

SGLT2i, including canagliflozin, dapagliflozin, empagliflozin, and ertugliflozin, were evaluated in a propensity-matched, multicenter retrospective cohort study within the Veteran Affairs Health Care System (VAHCS). Our cohort included patients from 125 distinct VA clinical sites, referred to as station numbers (STA3Ns), of which 104 had patients admitted to the ICU. The sample included adults admitted for sepsis with or without prior use of SGLT2i use. SGLT2i use prior to hospitalization was defined as having at least three outpatient prescription fills in the year before admission. For patients with fewer than three fills during that period, the last fill was required to be within 180 days prior to hospitalization (eFig. S1). Data were extracted from Medical Statistical Analysis System (MedSAS) and Corporate Data Warehouse (CDW) files in the Veterans Affairs Informatics and Computing Infrastructure. Institutional review board approval (University of California, San Francisco, and Veterans Affairs Central Institutional Review Board) for this research was obtained (IRB 10-03609).

Sepsis and septic shock were identified using ICD-10 codes (see supplement) [14,15]. Site specific infections were identified using ICD-10 codes recorded during the index hospitalization.

### Patient population

**Fig. 1:**
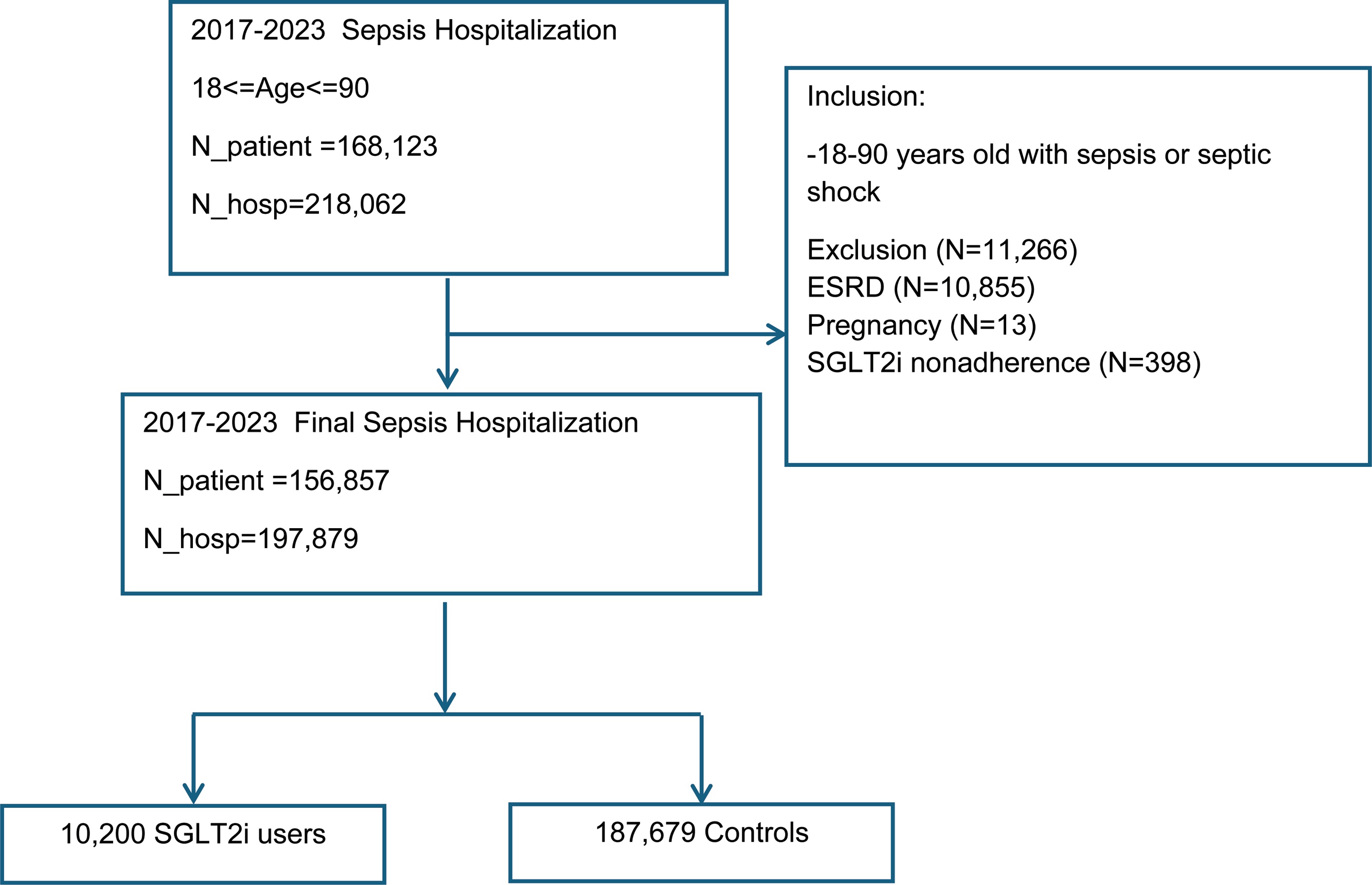
Flow chart demonstrating the distribution of the study cohort according to inclusion and exclusion criteria. SGLT2i: sodium-glucose cotransporter 2 inhibitors; ICU: intensive care unit; ESRD: end-stage renal disease; COVID: coronavirus; DM: diabetes mellitus; CHF: chronic heart failure; CKD: chronic kidney disease

The sample included patients ≥18 years old admitted for sepsis or septic shock between January 2017 and December 2023. Exclusion criteria included nonadherence to SGLT2i (<3 fills or >180-day gap), and contraindicated conditions (e.g., pregnancy, end-stage renal disease). Patients with missing data (e.g., race) were not excluded; instead, these values were imputed (Fig. 1). COVID-19 status was defined based on the presence of any documented positive test results in the VA Corporate Data Warehouse. The unit of analysis was individual hospitalizations rather than unique patients.

### Primary and secondary outcomes

The primary outcome of this study is the occurrence of all-cause mortality within 90 days after admission.

Secondary outcomes include major adverse cardiovascular events (MACE), acute kidney injury (AKI) within seven days of hospital admission, major adverse kidney events within 30 days of admission (MAKE-30), euglycemic diabetic ketoacidosis (EDKA), and hospital length of stay. MACE were defined as a composite of acute myocardial infarction, stroke, and cardiovascular death, identified using ICD-10 codes. MACE and mortality outcomes were assessed within 90 days following the admission date, including events occurring during the hospitalization. AKI is defined by the Kidney Disease Improving Global Outcomes (KDIGO) [16] criteria as an increase in serum creatinine (SCr): either an increase of ≥0.3 mg/dL (≥26.5 µmol/L) within 48 h or an increase in SCr to ≥1.5 times baseline within the prior 7 days. Severe AKI is classified as KDIGO stage 2–3, based on creatinine values or the need for renal replacement therapy. Baseline SCr was defined as the value measured closest to and prior to admission date, or, if not available, the first SCr measurement during hospitalization. MAKE-30 is defined as a composite of: initiation of renal replacement therapy identified by CPT/HCPCS codes (90935–90937 and 90945–90947) or ICD-10 codes (N18.6, Z49), death within 30 days, and creatinine at the measurement closest to day 30 being ≥1.5 times the baseline value. For patients discharged prior to 30 days, outpatient serum creatinine measurements were used to assess the MAKE-30 components. If no outpatient laboratory values were available after discharge, the last available inpatient or outpatient creatinine value within the 30-day window was used. EDKA was explored as a safety outcome and is defined by ICD-10 codes.

### Statistical analysis

**Fig. 2:**
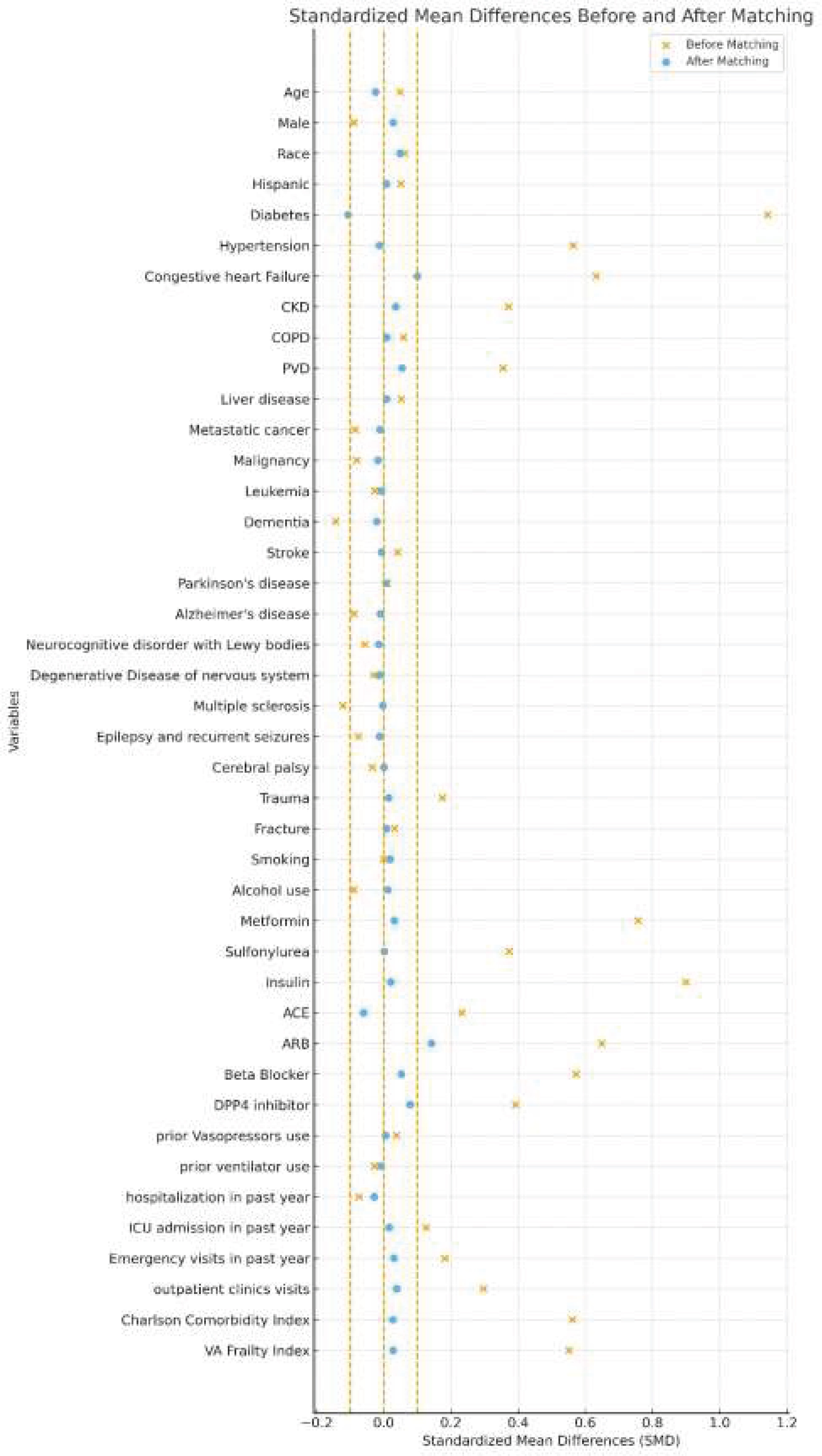
Standardized mean differences before and after 1:4 matching of baseline characteristics. This figure demonstrates that most of the baseline variables have SMD ≤ 0.1, indicating a proper balance between the groups. The only outlier outside of our range is ARB. CKD: chronic kidney disease; COPD: chronic obstructive pulmonary disease; PVD: peripheral vascular disease. ACE: angiotensin-converting-enzyme inhibitors; ARB: angiotensin receptor blockers; DPP4 inhibitor: dipeptidyl peptidase 4 inhibitor. VA: Veteran Affairs

SGLT2i users and non-users were compared after propensity score 1–4 matching (PSM) [17]. Baseline characteristics including demographics, comorbidities, and medications were compared between SGLT2i users and non-users before and after PSM. The logistic regression propensity score model included adjustment for a comprehensive set of variables: demographics, comorbidities, prior healthcare utilization (emergency department visit, number of outpatient visits, ventilator status), and the VA Frailty Index [18]. The Charlson Comorbidity Index (CCI) was used to numerically assess the burden of chronic comorbidities. Categorical variables were presented as numbers and percentages, while continuous variables were presented as mean and standard deviation. Standardized mean differences (SMD) were calculated to evaluate the balance of baseline covariates, with SMD ≤ 0.1 indicating good matching [19] (Fig. 2).

The final matched analytic cohort included 10,200 SGLT2i users and 37,785 controls, yielding an average match ratio of approximately 1:3.7 due to limitations in covariate overlap and data availability. Despite this, the matching preserved strong covariate balance and maintained statistical power.

Because a simple logistic model can be misspecified when important non-linearities or interactions are omitted, we conducted a sensitivity analysis using SuperLearner to estimate the propensity score and perform matching (1:4). SuperLearner is an ensemble method that uses cross-validation to learn an optimal convex combination of candidate algorithms [20]. Our library included a main-effects generalized linear model (GLM), a GLM with pairwise interactions, penalized logistic regression, random forests, a feed-forward neural network, and gradient boosting.

The primary goal was to investigate the risk of 90-day mortality, MACE, AKI in the cohort compared to matched controls. Odds ratios (OR) for these outcomes were independently calculated and presented with 95% confidence intervals (CI). We also calculated the E-value to assess the robustness of our findings to unmeasured confounding. The E-value represents the minimum strength of association that an unmeasured confounder would need to have with both the exposure (SGLT2i use) and the outcome (90-day mortality) to fully explain the observed association [21,22]. Subgroup analyses examined ICU vs. non-ICU cases, as well as patients with diabetes, congestive heart failure (CHF), and chronic kidney disease (CKD) and prior coronavirus infection (COVID). In addition to AKI, we evaluated MAKE-30.

A post hoc power calculation was conducted for the primary composite outcome of MACE and 90-day mortality in the matched cohort.

## Results

### Baseline characteristics

Among 197,879 eligible patients, 5.2% were SGLT2i users and 94.8% were non-users (Table 1). Patients were older than 65 years of age (mean [SD]: 69.5 [11.6]), predominantly male (95.4%), and White (71.0%). Only 2.1% of hospitalizations involved patients on SGLT2i therapy. Among these, just 1.6% (n = 3,173/197,897) were patients who had been on SGLT2i before admission and continued treatment during hospitalization. Similarly, only 1.3% of ICU (n = 580/43,391) admissions involved patients who continued the treatment. The final matched cohort included 47,985 patients: 10,200 SGLT2i users and 37,785 non-users (Table 1). The median duration of inpatient therapy was 3 days (IQR: 1–7). Patients in the SGLT2i group had on average 1.36 hospitalizations (IQR: 1–2), while those in the non-user group had on average 1.71 hospitalizations (IQR: 1−1). The most common comorbidities included diabetes, hypertension, CHF, and CKD. Most patients were taking insulin (32.8%), beta-blockers (46.7%) and angiotensin-converting enzyme inhibitor (ACEi) (32.2%) prior to admission (Table 1).

**Table 1:** Baseline and hospitalization characteristics of the study population.

	Full Cohort				1:4 Matched Cohort			
	Non-gliflozin N = 187,679	Gliflozin N = 10,200	Overall N = 197,879	SMD	Non-gliflozin N = 37,785	Gliflozin N = 10,200	Overall N = 47,985	SMD
Demographics								
Age, Mean (SD, IQR)	69.5 (11.7, 64−77)	70.0 (9.1, 64−76)	69.5 (11.6, 64−77)	0.049	70.2 (9.4, 65−76)	70.0 (9.1, 64−76)	70.2 (9.4, 65−76)	−0.025
Male, n (%)	178884 (95.3)	9950 (97.5)	188834 (95.4)	−0.0895	36759 (97.3)	9950 (97.5)	46709 (97.3)	0.028
Race, n (%)				0.0629				0.0483
American Indian or Alaska Native	1615 (0.9)	69 (0.7)	1684 (0.9)		280 (0.7)	69 (0.7)	349 (0.7)	
Asian	991 (0.5)	77 (0.8)	1068 (0.5)		235 (0.6)	77 (0.8)	312 (0.7)	
Black or African American	39318 (20.9)	1978 (19.4)	41296 (20.9)		7451 (19.7)	1978 (19.4)	9429 (19.6)	
Native Hawaiian or Other Pacific Islander	1448 (0.8)	101 (1)	1549 (0.8)		362 (1)	101 (1)	463 (1)	
Unknown	11202 (6)	591 (5.8)	11793 (6)		2071 (5.5)	591 (5.8)	2662 (5.5)	
White	133105 (70.9)	7384 (72.4)	140489 (71)		27386 (72.5)	7384 (72.4)	34770 (72.5)	
Hispanic	15940 (8.5)	1016 (10)	16956 (8.6)	0.0507	3678 (9.7)	1016 (10)	4694 (9.8)	0.0076
Comorbidity, n (%)								
Diabetes	84793 (45.2)	9317 (91.3)	94110 (47.6)	1.1421	35552 (94.1)	9317 (91.3)	44869 (93.5)	-0.1059
Hypertension	136158 (72.5)	9487 (93)	145645 (73.6)	0.563	35274 (93.4)	9487 (93)	44761 (93.3)	-0.0137
Congestive heart failure	44471 (23.7)	5406 (53)	49877 (25.2)	0.6321	18151 (48)	5406 (53)	23557 (49.1)	0.0994
CKD	43543 (23.2)	4098 (40.2)	47641 (24.1)	0.3711	14538 (38.5)	4098 (40.2)	18636 (38.8)	0.0348
COPD	66352 (35.4)	3892 (38.2)	70244 (35.5)	0.0582	14270 (37.8)	3892 (38.2)	18162 (37.8)	0.008
PVD	40389 (21.5)	3820 (37.5)	44209 (22.3)	0.3548	13160 (34.8)	3820 (37.5)	16980 (35.4)	0.0546
Liver disease	25603 (13.6)	1580 (15.5)	27183 (13.7)	0.0524	5743 (15.2)	1580 (15.5)	7323 (15.3)	0.0081
Metastatic cancer	15052 (8)	598 (5.9)	15650 (7.9)	−0.085	2320 (6.1)	598 (5.9)	2918 (6.1)	−0.0117
Malignancy	54305 (28.9)	2588 (25.4)	56893 (28.8)	−0.0802	9876 (26.1)	2588 (25.4)	12464 (26)	−0.0175
Leukemia	4099 (2.2)	184 (1.8)	4283 (2.2)	−0.0272	719 (1.9)	184 (1.8)	903 (1.9)	−0.0073
Dementia	20764 (11.1)	710 (7)	21474 (10.9)	−0.1436	2829 (7.5)	710 (7)	3539 (7.4)	−0.0203
Stroke	21484 (11.4)	1302 (12.8)	22786 (11.5)	0.0404	4913 (13)	1302 (12.8)	6215 (13)	−0.0071
Parkinson’s disease	98 (0.1)	8 (0.1)	106(0.1)	0.0103	24 (0.1)	8 (0.1)	32 (0.1)	0.0056
Alzheimer’s disease	3891 (2.1)	101 (1)	3992 (2)	−0.0883	413 (1.1)	101 (1)	514 (1.1)	−0.0101
Neurocognitive disorder with Lewy bodies	1489 (0.8)	37 (0.4)	1526 (0.8)	−0.0568	173 (0.5)	37 (0.4)	210 (0.4)	−0.0149
Degenerative disease of nervous system	1296 (0.7)	47 (0.5)	1343 (0.7)	−0.0304	206 (0.5)	47 (0.5)	253 (0.5)	−0.0119
Multiple sclerosis	2854 (1.5)	36 (0.4)	2890 (1.5)	−0.1214	140 (0.4)	36 (0.4)	176 (0.4)	−0.0029
Epilepsy and recurrent seizures	9329 (5)	353 (3.5)	9682 (4.9)	−0.0752	1396 (3.7)	353 (3.5)	1749 (3.6)	−0.0126
Cerebral palsy	109 (0.1)	0 (0)	109 (0.1)	−0.0341	0 (0)	0 (0)	0 (0)	0
Trauma	77710 (41.4)	5109 (50.1)	82819 (41.9)	0.1749	18655 (49.4)	5109 (50.1)	23764 (49.5)	0.0143
Fracture	15735 (8.4)	948 (9.3)	16683 (8.4)	0.0321	3429 (9.1)	948 (9.3)	4377 (9.1)	0.0076
Smoking	45859 (24.4)	2491 (24.4)	48350 (24.4)	−0.0003	8926 (23.6)	2491 (24.4)	11417 (23.8)	0.0187
Alcohol use	25863 (13.8)	1106 (10.8)	26969 (13.6)	−0.0895	3959 (10.5)	1106 (10.8)	5065 (10.6)	0.0118
VA frailty index [Mean (SD)]	0.2 (0.14)	0.3 (0.14)	0.2 (0.14)	0.5512	0.3 (0.14)	0.3 (0.14)	0.3 (0.14)	0.028
Charlson [Mean (SD)]	4.1 (3.48)	6.1 (3.33)	4.2 (3.50)	0.5618	6.0 (3.34)	6.1 (3.33)	6.0 (3.34)	0.0267
Medication, n (%)								
Metformin	40429 (21.5)	5716 (56)	46145 (23.3)	0.757	20591 (54.5)	5716 (56)	26307 (54.8)	0.0311
Sulfonylurea	17879 (9.5)	2356 (23.1)	20235 (10.2)	0.3737	8703 (23)	2356 (23.1)	11059 (23)	0.0015
Insulin	57651 (30.7)	7315 (71.7)	64966 (32.8)	0.8993	26751 (70.8)	7315 (71.7)	34066 (71)	0.0203
ACEi	59402 (31.7)	4365 (42.8)	63767 (32.2)	0.2321	17307 (45.8)	4365 (42.8)	21672 (45.2)	−0.0606
ARB	25201 (13.4)	4170 (40.9)	29371 (14.8)	0.649	12868 (34.1)	4170 (40.9)	17038 (35.5)	0.1414
Beta Blocker	85057 (45.3)	7382 (72.4)	92439 (46.7)	0.5717	26464 (70)	7382 (72.4)	33846 (70.5)	0.0516
DPP4 inhibitor	6605 (3.5)	1482 (14.5)	8087 (4.1)	0.3916	4480 (11.9)	1482 (14.5)	5962 (12.4)	0.079
Hospitalization Characteristics, n (%)								
LOS, [mean (SD), median (IQR)]	18.6 (75.54), 6 (3−13)	13.4 (30.62), 7 (4−14)	18.3 (73.91), 6 (3−13)	−0.0895	18.1 (67.06), 7 (4−14)	13.4 (30.62), 7 (4−14)	17.1 (61.19), 7 (4−14)	−0.0895
Vasopressors	12099 (6.4)	782 (7.7)	12881 (6.5)	0.0477	2492 (6.6)	782 (7.7)	3274 (6.8)	0.0416
Ventilator Status	7429 (4)	346 (3.4)	7775 (3.9)	−0.0301	1379 (3.6)	346 (3.4)	1725 (3.6)	−0.014
COVID ever positive	51357 (27.4)	4251 (41.7)	55608 (28.1)	0.3045	10968 (29)	4251 (41.7)	15219 (31.7)	0.2669
ICU Admission	41051 (21.9)	2340 (22.9)	43391 (21.9)	0.0256	8669 (22.9)	2340 (22.9)	11009 (22.9)	0.0000
Infection Source, n (%)								
Pneumonia	4117 (2.2)	114 (1.1)	4231 (2.1)	−0.0844	756 (2)	114 (1.1)	870 (1.8)	−0.0713
Soft tissue	65 (0)	4 (0)	69 (0)	0.0024	15 (0)	4 (0)	19 (0)	−0.0002
Catheter	4767 (2.5)	207 (2)	4974 (2.5)	−0.0342	890 (2.4)	207 (2)	1097 (2.3)	−0.0223
UTI	3121 (1.7)	82 (0.8)	3203 (1.6)	−0.0779	624 (1.7)	82 (0.8)	706 (1.5)	−0.077
Wound	1723 (0.9)	134 (1.3)	1857 (0.9)	0.0377	364 (1)	134 (1.3)	498 (1)	0.033
Biliary infection	295 (0.2)	16 (0.2)	311 (0.2)	−0.0001	71 (0.2)	16 (0.2)	87 (0.2)	−0.0075
Bacteremia	401 (0.2)	19 (0.2)	420 (0.2)	−0.0061	102 (0.3)	19 (0.2)	121 (0.3)	−0.0175
Abdominal	1847 (1)	84 (0.8)	1931 (1)	−0.017	353 (0.9)	84 (0.8)	437 (0.9)	−0.0119
Prior Health Utilization								
Prior vasopressors use	2075 (1.1)	156 (1.5)	2231 (1.1)	0.0372	556 (1.5)	156 (1.5)	712 (1.5)	0.0048
Prior ventilator use	3368 (1.8)	147 (1.4)	3515 (1.8)	−0.028	580 (1.5)	147 (1.4)	727 (1.5)	−0.0077
Hospitalization in past year	26233 (14)	1174 (11.5)	27407 (13.9)	−0.0741	4702 (12.4)	1174 (11.5)	5876 (12.2)	−0.0288
ICU admission in past year	19032 (10.1)	1453 (14.2)	20485 (10.4)	0.1257	5176 (13.7)	1453 (14.2)	6629 (13.8)	0.0158
Emergency visits in past year	139483 (74.3)	8346 (81.8)	147829 (74.7)	0.1821	30467 (80.6)	8346 (81.8)	38813 (80.9)	0.0305
Outpatient clinics visits [Mean (SD)]	65.0 (53.85)	80.5 (50.77)	65.8 (53.80)	0.2969	78.5 (53.80)	80.5 (50.77)	79.0 (53.18)	0.0379

SMD: standardized mean difference; SD: standard deviation; IQR: interquartile range; CKD: chronic kidney disease; COPD: chronic obstructive pulmonary disease; PVD: peripheral vascular disease; VA: Veteran Affairs; ACEi: angiotensin-converting enzyme inhibitors; ARB: angiotensin II receptor blocker; DPP4 inhibitor: dipeptidyl peptidase-4 inhibitor; LOS: length of stay; COVID: coronavirus; UTI: urinary tract infection; ICU: intensive care unit.

### Risk of 90-day mortality

**Fig. 3:**
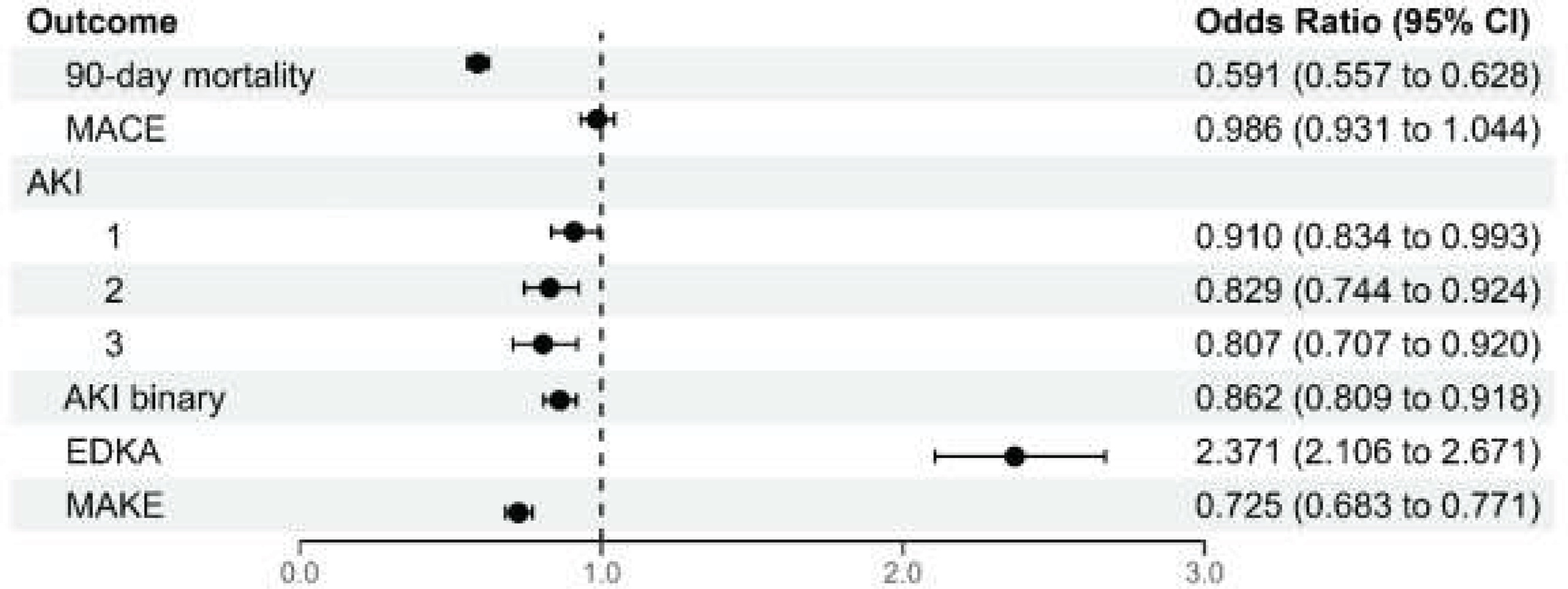
Clinical outcomes for the SGLT2i users and controls include 90-day mortality, MACE, AKI, EDKA, and MAKE. This forest plot demonstrates the difference between the overall cohort outcomes with the odds ratios and 95% confidence intervals. MACE: major adverse cardiovascular event; AKI: acute kidney injury; EDKA: euglycemic diabetic ketoacidosis, MAKE: major adverse kidney event

In the pre-matched population, 90-day mortality after admission was 14.2% among SGLT2i users and 21.7% among non-users. After propensity score matching, the 90-day mortality was significantly lower in the SGLT2i group compared to control (14.2% vs. 21.9%, p < 0.0001, Table 2). SGLT2i users were associated with 40.9% lower odds of 90-day all-cause mortality compared with non-users (OR = 0.591, 95% CI: 0.557−0.628) (Fig. 3). The E-value for the point estimate is 2.77 (and 2.56 for the CI), meaning an unmeasured confounder would need to be associated with both SGLT2i use and 90-day mortality by risk ratios of at least 2.77 (and 2.56 to move the CI to include the null) to explain away the association, which suggests the robustness of the estimated results against unmeasured confounding. This effect was also observed on subgroup analysis comparing ICU vs non-ICU patients, as well as those with and without diabetes, CHF, and CKD (eFig. S2). As a sensitivity analysis, we performed a Cox proportional hazards regression using time from admission to death as the outcome, censoring at 90 days. Results were consistent with our logistic regression findings (hazard ratio = 0.623, 95% CI: 0.589−0.658). Results were similar in the SuperLearner sensitivity analysis, which achieved well-balanced matches (eFig. S3). The odds ratio for 90-day mortality was 0.65 (95% CI, 0.611–0.691; eTable S1), with an E-value of 2.45 (2.25 for the CI).

**Table 2:** Outcomes and hospitalization characteristics.

	Full Cohort					1:4 Matched Cohort				
	Non-gliflozin N = 187,679	Gliflozin N = 10,200	Overall N = 197,879	p-value	SMD	Non-gliflozin N = 37,785	Gliflozin N = 10,200	Overall N = 47,985	p-value	SMD
Outcomes, n (%)										
90-day mortality	40693 (21.7)	1450 (14.2)	42143 (21.3)	<0.0001	−0.1955	8270 (21.9)	1450 (14.2)	9720 (20.3)	<0.0001	−0.2005
MACE	28571 (15.2)	1791 (17.6)	30362 (15.3)	<0.0001	0.0631	12811 (33.9)	2892 (28.4)	15703 (32.7)	<0.0001	−0.1201
AKI	24873 (13.4)	1388 (13.7)	26261 (13.4)	0.3594	0.0093	5844 (15.6)	1388 (13.7)	7232 (15.2)	<0.0001	−0.0524
AKI stage 1	11255 (6.1)	685 (6.8)	11940 (6.1)			2732 (7.3)	685 (6.8)	3417 (7.2)		
AKI stage 2	7545 (4.1)	421 (4.2)	7966 (4.1)			1843 (4.9)	421 (4.2)	2264 (4.7)		
AKI stage 3	6073 (3.3)	282 (2.8)	6355 (3.2)			1269 (3.4)	282 (2.8)	1551 (3.3)		
MAKE-30	34412 (18.3)	1491 (14.6)	35903 (18.1)	<0.0001	−0.1003	7215 (19.1)	1491 (14.6)	8706 (18.1)	<0.0001	−0.1198
EDKA	1971 (1.1)	457 (4.5)	2428 (1.2)	<0.0001	0.2103	733 (1.9)	457 (4.5)	1190 (2.5)	<0.0001	0.1445

SD: standardized mean difference; IQR: interquartile range; MACE: major adverse cardiovascular events; AKI: acute kidney injury; MAKE-30 major adverse kidney events within 30 days of admission; EDKA: euglycemic diabetic ketoacidosis.

### Risk of major adverse cardiovascular events

Overall, 17.7% patients experienced MACE, with 17.6% in the SGLT2i group and 17.8% in the control group. SGLT2i use was not associated with a significant reduction in the odds of MACE (OR = 0.986, 95% CI: 0.931–1.04) (Table 2, Fig. 3). Subgroup analysis revealed similar findings in both ICU and non-ICU patients, as well as among those with and without CHF and CKD (eFig. S2). Previously COVID infection status and non-diabetics appeared to be effect modifiers. SGLT2i use was associated with higher odds of MACE in patients who tested positive for COVID (OR = 1.12, 95% CI: 1.02–1.23), while in those who tested negative, SGLT2i use was linked to a reduced odds of MACE (OR = 0.935, 95% CI: 0.869–1.01). Similarly, SGLT2i use was associated with higher odds of MACE in non-diabetics (OR = 1.30, 95% CI: 1.06–1.58) compared to diabetics (OR = 0.964, 95% CI: 0.908–1.02).

### Risk of acute kidney injury

A total of 15.2% of the matched cohort experienced AKI, with 13.7% in the SGLT2i group and 15.6% in the control group (OR = 0.862, 95% CI: 0.809−0.918) (Fig. 3, Table 2). The mean baseline SCr was 1.68 mg/dL (SD: 1.31) in the SGLT2i group and 1.64 mg/dL (SD: 1.05) in the control group. SGLT2i use was associated with protective effects for stage 1 (OR = 0.910, 95% CI: 0.834−0.993), stage 2 (OR = 0.829, 95% CI: 0.744−0.924), and stage 3 (OR = 0.807, 95% CI: 0.707−0.920) AKI (Fig. 3). Subgroup analysis revealed a decreased risk of AKI among non-ICU patients, COVID-negative patients, patients with diabetes, patients with and without CHF, and patients with and without CKD (eFig. S2).

Overall, 18.1% of the full cohort experienced MAKE-30 (18.3% non-users; 14.6% SGLT2i users). After matching, 18.1% experienced MAKE-30 (19.1% non-users; 14.6% SGLT2i users). SGLT2i use was associated with a significantly lower risk of MAKE-30 (OR = 0.725, 95% CI: 0.683−0.771), corresponding to a 27.5% risk reduction compared with non-users following sepsis hospitalization.

Of note, the interaction between SGLT2i and angiotensin-converting enzyme inhibitor (ACEi) was not statistically significant for all-cause mortality, MACE, or AKI across all stages, indicating no meaningful interaction between the two classes (eTable S2). In contrast, the SGLT2i and angiotensin II receptor blocker (ARB) interaction was statistically significant for the primary outcome (OR = 1.16; 95% CI: 1.03–1.32), suggesting a modest increase in risk when both medications were used concurrently. When stratified by AKI stage, this association was significant only for stage 2 AKI (OR = 1.33; 95% CI: 1.07–1.65), and not for stages 1 or 3. These findings point to a potential interaction between SGLT2i and ARB use that may vary on the severity of kidney injury.

### Length of stay

The median length of stay for non-users was 6 days [IQR: 3−13] and 7 days [IQR: 4−14] for SGLT2i users.

### Risk of euglycemic diabetic ketoacidosis

A total of 1.2% of the full cohort had EDKA, with 4.5% in the SGLT2i group and 1.1% in the control group (Table 2). 2.5% of the matched cohort had EDKA (1.9% in non-users; 4.5% in SGLT2i users). SGLT2i use significantly increased the risk of EDKA (OR = 2.37, 95% CI: 2.11–2.67) (Fig. 3. eTable S3). Absolute risk difference is 2.54%. Subgroup analysis also confirmed a consistent increase in EDKA among SGLT2i users (eFig. S2). Notably, this increased risk was primarily observed among individuals with diabetes, suggesting that diabetes may be a key effect modifier.

### Impact of SGLT2i resumption after discharge

We performed a sensitivity analysis among SGLT2i survivors to explore the impact of resuming the medication after hospital discharge. Within 90 days post-discharge, 4,617 patients resumed SGLT2i therapy, with a median time to resumption of 29 days (IQR: 11–53 days). Among those patients, the median duration of medication supplied was 90 days (IQR: 90−90 days), with a mean of 92 days. After adjusting for vasopressor use, the need for invasive mechanical ventilation, dialysis, and ICU admission, patients who were continued on SGLT2i therapy after discharge had a significantly reduced risk of 90-day mortality (OR = 0.559, 95% CI: 0.524−0.595) and a significant reduction in the odds of AKI (OR = 0.825, 95% CI: 0.771−0.882) compared to those whose SGLT2i were discontinued (eTable S4).

Among prior SGLT2i users, resuming the medication 90 days after hospitalization was protective for 90-day mortality (OR = 0.23; 95% CI: 0.19−0.28) but was associated with a higher risk of MACE (OR = 1.27; 95% CI: 1.13–1.44); both outcomes were assessed 90 days after discharge. Resuming therapy was protective against 90-day mortality (2.62% among resumers and 10.46% among non-resumers). In contrast, the incidence of MACE was higher in the resume group (12.84%) than in the non-resume group (10.37%). In the matched cohort, only 692 patients initiated SGLT2i therapy within 90 days after discharge.

## Discussion

In this nationwide, multicenter, retrospective cohort study of adult veterans with sepsis, SGLT2i use prior to hospital admission was associated with a significantly lower risk of 90-day all-cause mortality and reduction in the odds of AKI. This finding suggests that SGLT2i therapy may confer potential organ protection prior to the development of sepsis. Although the incidence of MACE was lower among SGLT2i users, it was not statistically different.

Ninety-day mortality is a widely used endpoint in sepsis and cardiovascular outcome studies, as it captures both in-hospital deaths and early post-discharge mortality, which may reflect ongoing complications or subacute deterioration following the index hospitalization. Survival to discharge overlooks patients who die shortly thereafter, a limitation particularly relevant in older adults and those recovering from sepsis. By extending the follow-up window to 90 days, we aimed to capture the full burden of mortality attributable to both the acute illness and its sequelae. The lower mortality in patients with sepsis suggests that the protective effect observed in chronic cardiovascular disease and chronic kidney disease may extend to acute settings when patients develop sepsis. Organ protection may confer more physiological reserve in patients developing sepsis, increasing their chance of recovery. These findings align with one small retrospective study that has suggested lower inpatient mortality among diabetic patients with septic shock who were on a SGLT2i prior to admission [23].While SGLT2i’s benefits in chronic settings are well-documented, with mechanisms including reduced glycolysis and mitochondrial reactive oxygen species formation, reduced volume overload through osmotic diuresis and natriuresis [24,25], and enhanced lipolysis and ketone body production [26], the translation of these mechanisms to acute setting like sepsis remains uncertain.

While some studies raise concerns about an increased risk of AKI with SGLT2i use due to osmotic diuresis and hypovolemia [24,27], our findings suggest a protective association in the acute setting of sepsis. This may reflect renal protective mechanisms identified in chronic settings, such as reducing oxygen demand of proximal tubule to reabsorb sodium and glucose, decreasing inflammation and fibrosis, and lowering albuminuria [28,29] translating into an increased renal reserve. A meta-analysis of 13 trials with 90,413 participants found a 23% reduction in AKI risk with SGLT2i use (RR = 0.77, 95% CI: 0.70−0.84) in both diabetic and non-diabetic outpatient populations [30]. While our findings are directionally consistent, differences in study design and patient acuity preclude direct comparisons. Although discontinuation of SGLT2i at hospital admission can reverse their hemodynamic effects and lower serum creatinine, potentially influencing AKI classification, baseline serum creatinine values were well balanced between groups after matching. It is unlikely that withdrawal-related changes in creatinine explain the lower AKI incidence observed among SGLT2i users.

We also observed an increased risk of EDKA among SGLT2i users (OR = 2.371, 95% CI: 2.106–2.671), which appeared to be driven primarily by patients with diabetes (eFig. S2). This is consistent with known pharmacologic effects of SGLT2 inhibitors, including reduced insulin secretion and elevated glucagon levels [26]. Clinical guidelines acknowledge EDKA as a rare but serious complication and recommend holding SGLT2i during acute illness, surgery, or prolonged fasting [[26], [27], [28], [29]].

Although our study identified a mortality benefit associated with continued SGLT2i use during hospitalization, continuation in the hospital was uncommon in the ICU and among mechanically ventilated patients. Among those who received SGLT2i in the ICU, the median duration of inpatient therapy was only 3 days (IQR: 1–7). In critically ill patients, especially those on mechanical ventilation or nil per os (NPO), enteral administration of oral medications is challenging and often withheld due to concerns about impaired gastrointestinal absorption and risk of aspiration. Most available SGLT2i are oral tablets with no intravenous formulation, limiting their use in patients unable to tolerate enteral intake. Observed continuation in the ICU likely reflects patients who were recovering and able to tolerate oral intake during their hospital stay.

Until now, randomized trials of SGLT2i initiation in critically ill patients have not shown benefits on outcomes [11,31]. The DEFENDER trial analyzed 507 patients with at least one acute organ dysfunction randomized to either 10 mg of dapagliflozin or standard of care and revealed that the adjusted odds ratio for hierarchical composite of hospital mortality, initiation of kidney replacement therapy, and ICU length of stay was 1.01 (95% CI: 0.90–1.13), and the adjusted odds ratio accounted for sepsis, use of vasopressors and mechanical ventilation was 1.06 (95% CI: 0.76–1.52) [11]. Also, the DARE-19 trial analyzed 1250 patients with COVID-19 randomized to either 10 mg dapagliflozin or matched placebo and revealed no significant prevention of organ dysfunction or death in dapagliflozin (hazard ratio [HR]: 0.80, 95% CI: 0.58–1.10) and no significant difference in mortality (HR: 0.77, 95% CI: 0.52–1.16) [31]. A small case-control study involving eighteen ICU patients with type 2 diabetes who received 10 mg of empagliflozin found no significant association with ketoacidosis, worsening kidney function, or mortality [32]. These trials differ from our study in treatment timing, patient selection, and exposure duration. Collectively, these findings suggest that the benefits of SGLT2i may be observed in patients who are prolongedly exposed to the medication prior to admission, but not when started in the acute phase.

### 4.1Limitations

This observational study has several limitations. There is substantial potential for unmeasured confounding and confounding by indication. Despite propensity score matching to balance measured covariates, SGLT2i users may differ systematically from non-users that are not fully captured by dataset, including illness severity, glycemic control, frailty, and clinician treatment preferences. Causal inference cannot be established, and residual confounding is likely. The observed difference in eDKA incidence – a well-known side effect of SGLT2i- however suggests effective matching. Additionally, the observed effect size and direction are consistent with those reported in randomized trials conducted in other settings. A randomized trial would need to enroll nearly 3000 patients to show a similar effect size with a power of 90%. Additionally, the predominantly older, white male population may limit generalizability of our findings to more diverse or non-VA healthcare settings. Also, the use of administrative ICD-10 codes to identify site specific infections introduces known limitations. When sepsis is coded as the principal diagnosis, accompanying infection site codes are often omitted, especially when the infectious source is unclear or when sepsis is prioritized for billing. The low proportion of patients with coded infections in our cohort likely reflects this under coding, rather than a true absence of infections. While this limits the ability to analyze infection site specific outcomes, it does not impact the primary exposure or mortality outcome, which are less sensitive to coding limitations. In addition, analysis based on hospitalization rather than unique patients may introduce bias. Although we matched on hospitalization, the number of unique patients differs between groups, particularly in the non-gliflozin cohort. This means that mortality events may not be fully independent when patients contribute multiple admissions, and the proportion of unique patients represented in each group could influence the observed effect estimates. In the full cohort, there were 8,790 SGLT2i users and 148,067 non-SGLT2i users, while the matched cohort included 8,790 SGLT2i users and 33,001 non-SGLT2i users. Even though the average number of hospitalizations was similar between groups, this approach may slightly overrepresent patients with frequent admissions and should be interpreted with caution.

## Conclusions

In this retrospective, propensity score-matched cohort study of veterans hospitalized with sepsis, chronic use of SGLT2 inhibitor prior to admission was associated with a lower risk of 90-day mortality and acute kidney injury, suggesting an organ protection that may persist during acute illness. The observational nature of the analysis precludes definitive causal inference. While continuation of SGLT2i during hospitalization was rare, exploratory analyses suggest that patients who remained on therapy had similar favorable outcomes.

## CRediT authorship contribution statement

Justine Tang contributed to data analysis, interpretation and manuscript writing, tables, figures. Bocheng Jing contributed to the data extraction, statistical planning and analysis, manuscript writing, tables, figures, and review. Krystal Karunungan contributed to the design and writing. Anusha Badathala contributed to data access and management. Arthur Wallace contributed to data access, study design, data analysis, and manuscript review. Matthieu Legrand designed and supervised the study, contributed to the data interpretation and writing of the manuscript and took responsibility for the integrity and accuracy of the data analysis. All authors reviewed and validated the final version of the manuscript.

## Funding

Dr. Justine Tang is supported by the National Institutes of Health (Bethesda, Maryland) under award number T32GM008440. Dr. Matthieu Legrand is supported by grant R01-GM151494-01 and R01DK139484-01 from the NIH. The funders had no role in the design and conduct of the study; collection, management, analysis, and interpretation of the data; preparation, review, or approval of manuscript; and decision to submit the manuscript for publication. The authors have no other conflicts of interest to declare. Dr Legrand is an associate editor of Annals of Intensive Care.

## Availability of data and materials

The datasets used and/or analyzed during the current study can not be shared.
