# Supplementary material for: Association Between Gliflozins Use and Outcomes in Adults with Sepsis: A Multicenter Retrospective Cohort Study Among Veterans
Source: Ann Intensive Care. 2026 Jan 16;16:100021. doi: 10.1016/j.aicoj.2025.100021 (PMC12934444; doi:10.1016/j.aicoj.2025.100021)
Supplement: Supplementary file 1 [file mmc1.docx]

**Association Between Gliflozins Use and Outcomes in Adults with Sepsis: A Multicenter Retrospective Cohort Study Among Veterans**

Justine Tang, MD (1), Bocheng Jing, MS (2, 3), Krystal Karunungan, MD (1), Anusha Badathala, MD (3), Arthur Wallace, MD, PhD (1, 3), Matthi­­­eu Legrand, MD, PhD (1)

**eFigure 1. Timeline Illustrating the Criteria for SGLT2i Adherence Prior to Hospitalization**

**eFigure 2. Forest Plots for Prespecified Subgroups**

**eFigure 3. Standardized Mean Differences Before Matching, After Matching, and After SuperLearner Post-Matching**

**eTable 1. Post-Matching Results for Primary and Secondary Outcomes**

**eTable 2. Interaction Effects of SGLT2i and RAS Inhibitors on Outcomes**

**eTable 3. Distribution of SGLT2i Prescription and Incidence of EDKA**

**eTable 4. After Adjusting for Hospitalization Characteristics**

**ICD-10 codes**

**eFigure 1. Timeline Illustrating the Criteria for SGLT2i Adherence Prior to Hospitalization**

**
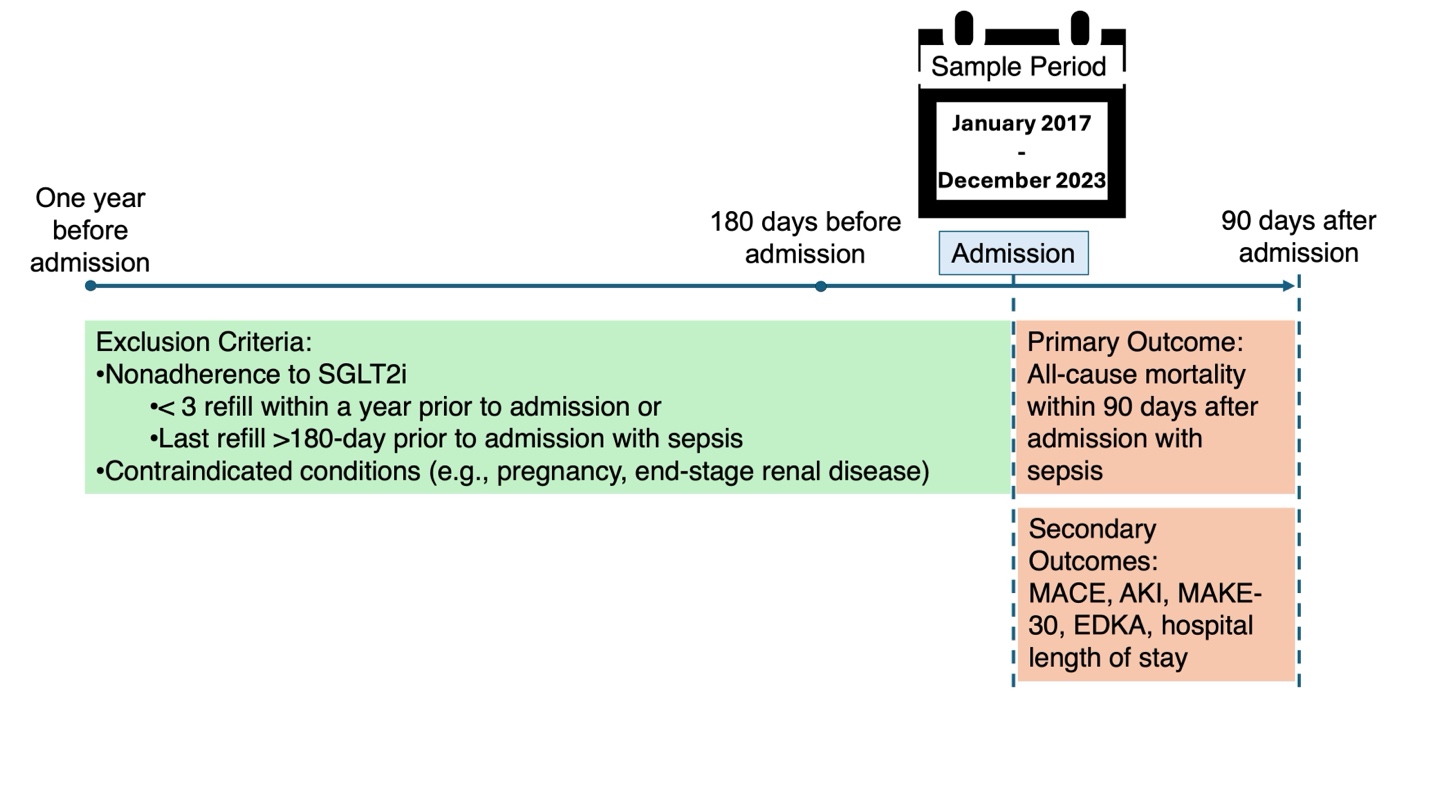
**

This is a timeline showing SGLT2i adherence prior to hospitalization. SGLT2i use prior to hospitalization is defined as having at least three outpatient prescription fills in the year before admission or must have their last fill within 180 days prior to hospitalization. Primary outcome is all-cause mortality within 90 days after admission with sepsis. Secondary outcomes are MACE, AKI, MAKE-30, EDKA, and hospital length of stay.

SGLT2i: sodium-glucose cotransporter-2 inhibitors
MACE: major adverse cardiovascular event; AKI: acute kidney injury; MAKE-30: major adverse kidney events within 30 days of admission; EDKA: euglycemic diabetic ketoacidosis

**eFigure 2. Forest Plots for Prespecified Subgroups**


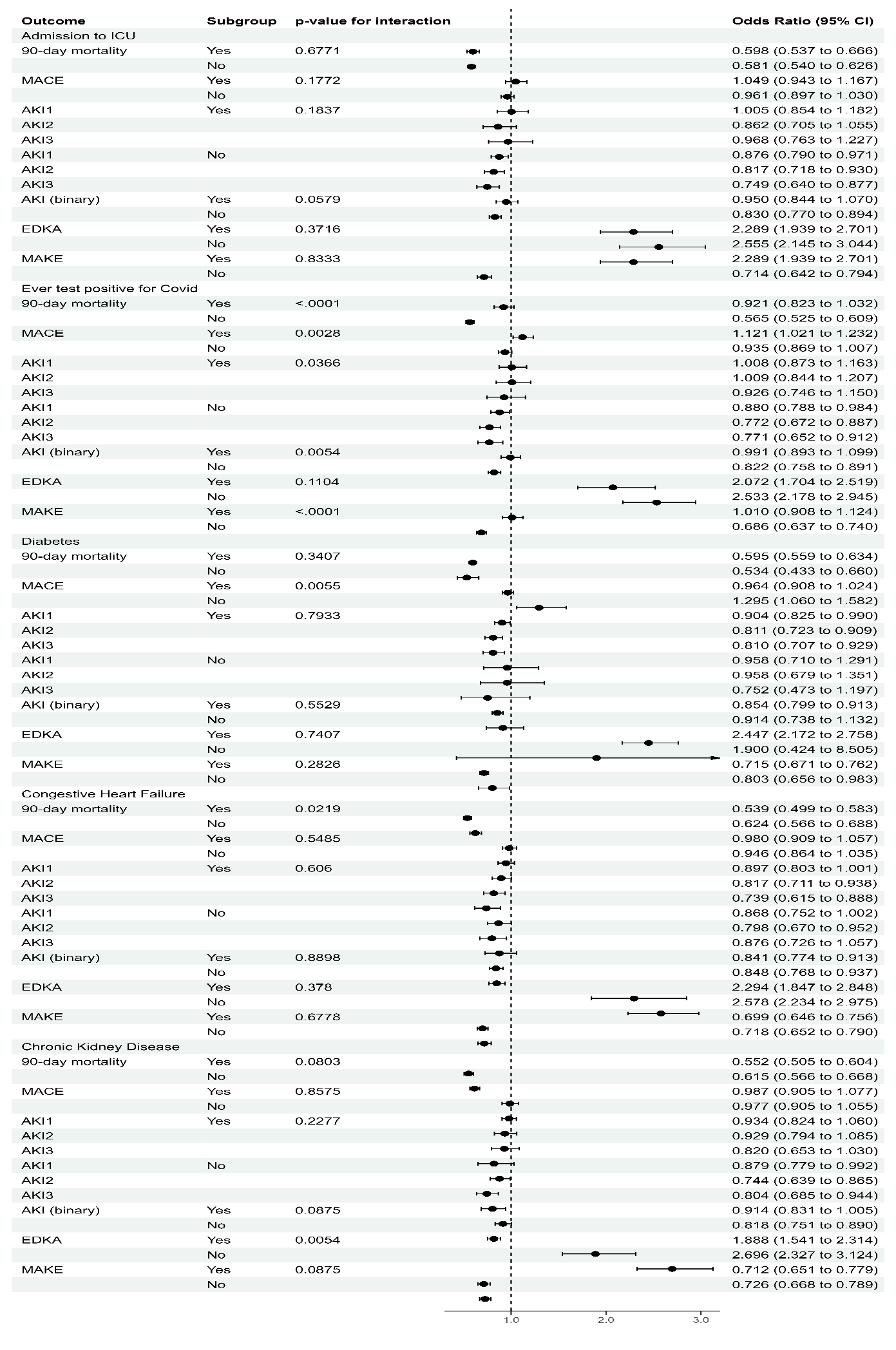


Forest plots for prespecified subgroups (ICU admission, prior exposure to COVID, diabetes mellitus, heart failure, chronic kidney disease) showing odds ratios (95% CI) and tests for interactions for the outcomes of 90-day mortality, MACE, AKI, EDKA, and MAKE.

MACE: major adverse cardiovascular event; AKI: acute kidney injury; EDKA: euglycemic diabetic ketoacidosis; MAKE: major adverse kidney event; ICU: intensive care unit; COVID: coronavirus; CI: confidence interval

**eFigure 3. Standardized Mean Differences Before Matching, After Matching, and After SuperLearner Post-Matching**

**
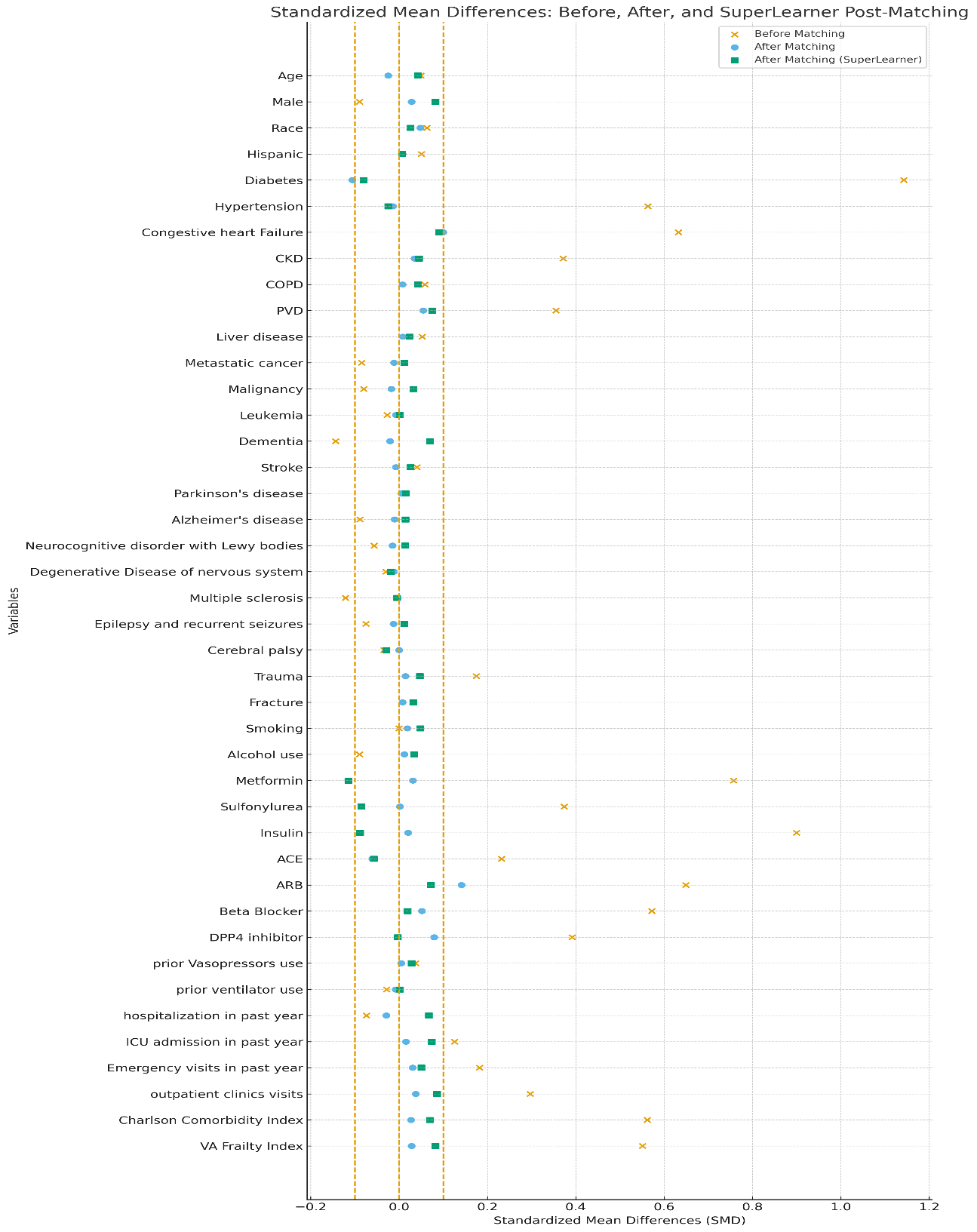
**

**eTable 1. Post-Matching Results for Primary and Secondary Outcomes**

|  | **1:4 PS Matching with Superlearner** | | |
| --- | --- | --- | --- |
| **Outcome** | **OR** | **Lower** | **Upper** |
| 90-day mortality | 0.65 | 0.611 | 0.691 |
| MACE | 1.046 | 0.987 | 1.108 |
| AKI |  |  |  |
| 1 | 0.922 | 0.845 | 1.006 |
| 2 | 0.851 | 0.763 | 0.949 |
| 3 | 0.801 | 0.702 | 0.914 |
| AKI binary | 0.864 | 0.811 | 0.921 |
| MAKE-30 | 0.743 | 0.699 | 0.79 |
| EDKA | 2.233 | 1.983 | 2.515 |
| MACE: major adverse cardiovascular events  AKI: acute kidney injury  MAKE-30: major adverse kidney event within 30 days  EDKA: euglycemic diabetic ketoacidosis  OR: odds ratio | | | |

**eTable 2. Interaction Effects of SGLT2i and RAS Inhibitors on Outcomes**

| Parameter | OR | Lower | Upper | Parameter | OR | Lower | Upper |
| --- | --- | --- | --- | --- | --- | --- | --- |
| Mortality | | | | | | | |
| SGLT2i | 0.571 | 0.527 | 0.620 | SGLT2i | 0.552 | 0.510 | 0.598 |
| ACEi | 0.987 | 0.940 | 1.037 | ARB | 0.990 | 0.941 | 1.042 |
| SGLT2i*ACEi | 1.070 | 0.946 | 1.210 | SGLT2i*ARB | 1.164 | 1.028 | 1.317 |
| MACE | | | | | | | |
| SGLT2i | 1.019 | 0.943 | 1.101 | SGLT2i | 0.921 | 0.853 | 0.994 |
| ACEi | 1.125 | 1.067 | 1.187 | ARB | 1.121 | 1.062 | 1.183 |
| SGLT2i*ACEi | 0.930 | 0.828 | 1.044 | SGLT2i*ARB | 1.106 | 0.984 | 1.243 |
| AKI | | | | | | | |
| SGLT2i (AKI= 1) | 0.965 | 0.859 | 1.083 | SGLT2i (AKI= 1) | 0.878 | 0.782 | 0.987 |
| SGLT2i (AKI= 2) | 0.881 | 0.764 | 1.016 | SGLT2i (AKI= 2) | 0.748 | 0.644 | 0.869 |
| SGLT2i (AKI= 3) | 0.879 | 0.740 | 1.044 | SGLT2i (AKI= 3) | 0.757 | 0.639 | 0.897 |
| ACEi (AKI= 1) | 1.098 | 1.015 | 1.187 | ARB (AKI= 1) | 1.136 | 1.048 | 1.232 |
| ACEi (AKI= 2) | 1.043 | 0.950 | 1.146 | ARB (AKI= 2) | 1.112 | 1.008 | 1.226 |
| ACEi (AKI= 3) | 1.092 | 0.976 | 1.222 | ARB (AKI= 3) | 1.006 | 0.896 | 1.129 |
| SGLT2i*ACEi (AKI= 1) | 0.880 | 0.738 | 1.049 | SGLT2i*ARB (AKI= 1) | 1.122 | 0.941 | 1.338 |
| SGLT2i*ACEi (AKI= 2) | 0.868 | 0.696 | 1.082 | SGLT2i*ARB (AKI= 2) | 1.327 | 1.066 | 1.652 |
| SGLT2i*ACEi (AKI= 3) | 0.823 | 0.630 | 1.074 | SGLT2i*ARB (AKI= 3) | 1.087 | 0.833 | 1.418 |
| OR: odds ratio  SGLT2i: sodium-glucose cotransporter-2 inhibitors  ACEi: angiotensin-converting enzyme inhibitors  ARB: angiotensin II receptor blockers  MACE: major adverse cardiovascular event  AKI: acute kidney injury | | | | | | | |


**eTable 3. Distribution of SGLT2i Prescription and Incidence of EDKA**

| SGLT2i | No EDKA (N=457) | Yes EDKA (N=9743) | Total (N=10200) |
| --- | --- | --- | --- |
| Canagliflozin | 6 (0.1%) | 0 (0%) | 6 (0.1%) |
| Dapagliflozin | 10 (0.1%) | 0 (0%) | 10 (0.1%) |
| Empagliflozin | 9727 (99.8%) | 457 (100%) | 10184 (99.8%) |
| SGLT2i: sodium-glucose cotransporter-2 inhibitors  EDKA: euglycemic diabetic ketoacidosis | | | |

This table shows the distribution of SGLT2i and incidence of EDKA among Veterans Affairs patients.

**eTable 4. After Adjusting for Hospitalization Characteristics**

| Outcome | OR | Lower | Upper |
| --- | --- | --- | --- |
| 90-day mortality | 0.559 | 0.524 | 0.595 |
| MACE | 0.976 | 0.922 | 1.034 |
| AKI |  |  |  |
| 1 | 0.883 | 0.808 | 0.965 |
| 2 | 0.787 | 0.704 | 0.88 |
| 3 | 0.742 | 0.647 | 0.852 |
| AKI binary | 0.825 | 0.771 | 0.882 |
| MAKE-30 | 0.674 | 0.632 | 0.719 |
| EDKA | 2.337 | 2.074 | 2.632 |

MACE: major adverse cardiovascular events

AKI: acute kidney injury

MAKE-30: major adverse kidney events within 30 days of admission

EDKA: euglycemic diabetic ketoacidosis

OR: odds ratio

This model represents the analysis after adjusting for hospitalization characteristics, focusing on the primary and secondary outcomes: 90-day mortality, MACE, AKI, MAKE-30, and EDKA. The odds ratio (OR), along with the lower and upper bounds of the 95% confidence intervals for each outcome, are presented.

**ICD-10 codes**

Inclusion:

Sepsis: A40, A41, R65.20

Septic shock: R65.21

Exclusion:

Pregnancy: Z33 and Z34

Lactation: Z39.1

End stage renal disease: N18.5, N18.6, I12.0, I13.2, I13.11, Z99.2

Primary outcomes:

AMI: I21-I22

Stroke: I60-I69

Secondary outcomes:

AKI: S37.0, N17, N17.0, N17.1, N17.2, N17.8, N17.9

EDKA: E0910, E1110

Additional variables:

Ventilation: Z99.1, Z99.11, J95.8

Pneumonia: J12-18, Z87.01

Soft tissue infection: M70, L08.89, L08.9

Catheter infection: T80.21, T83.518, T80.212, T80.218, T80.219, T83.511

UTI: N39.0

Wound infection: A49, T81.4, S11, S81, S31, S51, S71, S61, S41, S91, S01, S21

Biliary infection: K65, K81, K83, K85, K57, K65.1, K35-37

Bacteremia: R78.81, A49.9

Metastatic Cancer: C77-C79

Malignancy: C00-C96

Leukemia: C91-C95

Dementia: F02, F03

Stroke: I60-I63, I69

Parkinson’s disease: G20

Alzheimer’s disease: G30.9

Neurocognitive disorder with Lewy bodies: G31.83

Degenerative disease of nervous system: G31.9

Multiple sclerosis: G35

Epilepsy and recurrent seizures: G40

Cerebral palsy: G80

Trauma: S00-T88

Fracture: M84, S02, S12, S22, S32, S42, S52, S62, S72, S82, S92

Smoking: Z72.0, F17.200, F17.210, F17.220, F17.290

Alcohol use: F10.0, F10.10, F10.20
